# The boy who refused an IV: a case report of subcutaneous clodronate for bone pain in a child with Ewing Sarcoma

**DOI:** 10.1186/1752-1947-1-7

**Published:** 2007-03-21

**Authors:** Harold Siden

**Affiliations:** 1 Dept. of Pediatrics, University of British Columbia, and Canuck Place Children's Hospice, Vancouver, British Columbia, Canada

## Abstract

**Background:**

Bone pain in malignancy can be challenging to treat. Bisphosphonates have been found to be useful in adults with bone pain, but there are no reports of their use in children for this indication. In pediatric palliative medicine there are hurdles in translating knowledge gained primarily in adult studies into application in children. Obstacles exist in initially determining whether the evidence supports using a drug in children, and once a drug is chosen, then determining the optimal route of delivery. There is very little data to guide pediatric practitioners in this situation.

**Case Presentation:**

A 9 year old boy with disseminated Ewing Sarcoma presented with extremity pain not responsive to a combination of opiates, gabapentin and non-steroidal anti-inflammatory drugs. Clodronate, a bisphosphonate, was added to the regimen to treat bone pain. It was given subcutaneously every 4 weeks with a good response and no side effects.

**Conclusion:**

This case report describes the use of a bisphosphonate, clodronate, given subcutaneously to a child with Ewing sarcoma with effective relief of bone pain. It describes how the care team encountered the challenges inherent in translating adult therapy into a pediatric regimen. Furthermore the report details how a regimen was developed to address this child's concerns regarding medication administration. Further effort needs to be made at finding solutions to address the lack of good evidence for pediatric palliative therapies.

## Background

This report describes the use of subcutaneous clodronate to treat malignancy-related bone pain in a child. Pain is a significant problem in children with cancer, and treating it poses several challenges. One challenge for clinicians treating difficult pain in children is the lack of an evidence base and the frequent off-label use of medications. Treatment approaches and dose estimates are borrowed from adult medicine. A second challenge is to bridge to the child's developmental stage when devising an acceptable treatment. Developmentally appropriate behaviors and attitudes on the part of the child may be viewed by clinicians as difficult "issues". These may include taste preferences for medications, reluctance to swallow pills or refusal to have IV lines. Children may oppose particular treatment approaches even when "it is for their own good". This case report uses a simple example – the use of a previously unreported medication by a novel route – to illustrate the significant obstacles faced by clinicians providing pediatric palliative care, and to illustrate the processes often followed when devising treatment approaches.

## Case Presentation

The patient was a 9 year old boy with Ewing Sarcoma initially diagnosed just before his 6^th ^birthday. He underwent peripheral blood stem cell transplant. One year later he presented with recurrent disseminated disease and restarted chemotherapy and received radiation. He was also referred to the pediatric hospice program.

His symptoms included lower extremity paralysis and significant weakness of the upper extremities. He developed a partial gastric outlet obstruction, which later resolved to the point that he was able to resume oral feeding, but had difficulty taking oral medications. At the time of treatment his weight was 23 kg.

He had multiple pain complaints including headache, back pain and generalized arthralgia/myalgia. There was a particularly troubling bone pain involving his forearms, wrists and hands bilaterally. The arm pain was unpredictable, episodic and intense with aching and burning qualities. There were no shooting pains or electrical shock sensations, nor was there hyperalgesia or allodynia. Pain was assessed by verbal self-report as he did not want to use any of the standardized childhood pain scales, and by the nurse's assessment using the Canuck Place Comfort Assessment Tool.

His pain was treated with continuous subcutaneous or transdermal fentanyl at 75 micrograms/hour. He had hydromorphone for breakthrough pain, 3 milligrams subcutaneously as needed every hour. Adjuvant analgesics included round-the-clock gabapentin and naproxen. He received lorazepam as needed and ondansetron and nabilone for nausea. PEG 3350 and docusate were used as laxatives. The above regimen achieved satisfactory analgesia for all his pains except for bone pain in the arms.

The clinical team opted to try a bisphosphonate class drug for the bone pain. Selecting a medication route was a challenge; an indwelling vascular access device had been removed several weeks previously at the completion of chemotherapy. He refused to have a peripheral intravenous (IV) line placed because he found it uncomfortable and associated it with his previous unpleasant hospitalizations. Oral medications were considered, but with his recent history of gastric outlet obstruction, and his general reluctance (but not refusal) to take pills, this was not a reliable route. He refused rectal medications.

We therefore opted for a subcutaneous route, which was acceptable to him. A dose of 300 milligram of clodronate (~15 mg/kg) was prepared in 40 ml of normal saline (7.5 mg/ml). It was infused subcutaneously over an 8 hour time period and vital signs were monitored. There were no side effects and the infusion did not cause localized pain. There was no subsequent bone pain exacerbation. Serum calcium levels 4 days post infusion were 2.3 mmol/L with an albumin level of 28 g/L. Four days after the infusion he reported the onset of relief of the bone pain in his arms. Functionally he was improved, for example being able to use a video game controller without pain and manipulate board game pieces. The Comfort Assessment Tool provided additional information that pain was improved. He required no increases in his other analgesics; because they were providing adequate relief for other pains he experienced we did not decrease them either. The analgesic effect lasted almost 4 1/2 weeks, when he again reported intense bone pain, and the 300 mg dose was repeated, with good results 4 days later. Thereafter he received regular infusions every 4 weeks, prior to the expected recurrence of pain.

In order to minimize procedures in accordance with the emphasis on patient comfort, imaging studies were not obtained and laboratory studies were kept to a minimum. During this period he moved between home and hospice with readmissions for family care, respite and symptom management. He died peacefully at home 7 months after acceptance to the pediatric palliative program, and 19 days following his last clodronate infusion,

### Discussion: Bisphosphonate Therapy

When we decided to introduce a bisphosphonate because his extremity pain was not responsive to opiates or a series of adjuvant medications we were faced with the questions of which drug to use and by what route. Cancer-related bone pain is a particularly difficult symptom to treat. Bisphosphonates have gained acceptance as a standard approach to bone pain in adults. In the idealized scenario evidence exists to guide clinicians to the most effective drug with support for a dose and frequency. Similarly in the idealized scenario, the choice of medication route depends primarily on pharmacokinetic and pharmacy factors such as drug formulation. There is however a dearth of evidence regarding bisphosphonates in children, and controversy regarding dosing.

The major effect of bisphosphonates is to decrease recruitment and function of osteoclasts and thereby reduce bone turnover. Bisphosphonates act via multiple mechanisms at the cellular and molecular level [1]. The initial widespread use of bisphosphonates was in Paget's disease in the late 1970's and early 1980's. Bisphosphonate therapy was extended to the treatment of a number of other adult conditions such as cancer associated with bone destruction and hypercalcemia.

Noting the effectiveness in reducing bone turnover in a variety of adult conditions, bisphosphonates were then considered for use in children. Pamidronate is used routinely for children with Osteogenesis Imperfecta, given by IV. Since then bisphosphonate therapy has been trialed in a number of childhood diseases [2,3]. It should be noted, however, that none of the reports describe bisphosphonate use to treat malignancy-related pain in children. A recent publication describes treating two children with osteoporosis secondary to leukemia; however, there was no mention of pain [4].

### Exploring the evidence for subcutaneous bisphosphonates

Our patient expressed a strong preference not to have an IV. The oral route was not considered to be reliable. He expressed no objection to a subcutaneous line, having already experienced subcutaneous infusions for chemotherapy and analgesia. We therefore explored this route of administration for bisphosphonates.

We reviewed the literature on bisphosphonates [Medline 1966–2003] and engaged in email and telephone discussion with authors and expert clinicians in palliative medicine and pediatric endocrinology. [5]. The evidence was clear that pamidronate was safe and tolerable in children, but if given subcutaneously may have toxicities [6]. On the other hand, the best evidence for subcutaneous bisphosphonate infusions supported clodronate, but only for adults [7]. Zoledronic acid was also considered as it requires only a very brief IV infusion. There was no data, however, on its use in children; a subsequent report showed a high frequency of side effects in this population [8].

The only published report of clodronate use in children was by Zacharin and Cundy who gave it by IV infusion to a child with a genetic bone disease, osteoporosis pseudoglioma syndrome [9]. (An additional case report was published after we treated our patient [10]). The drug was well tolerated and helped to improve bone mineral density in their patient. These clinicians extrapolated from adult doses and used first 300 mg, then 600 mg, IV (10–20 mg/kg). [Personal communication. T. Cundy. Clodronate dosing [online]. Email to H. Siden (tcundy@auckland.ac.nz) 19 Jan 2004].

Because there were no reports on using clodronate to treat malignancy-related bone pain in children, we developed the regimen empirically. We relied on the dose reported by Zacharin and Cundy; in order to reduce fluid in the subcutaneous infusion, the maximum concentration described for drug preparation was used. The 8 hour infusion was chosen to minimize localized swelling; a shorter time period may work as well. We closely monitored our patient following the first two infusions and determined that 300 mg repeated every 4 weeks appeared to prevent pain recurrence.

## Observations

The first observation is that clodronate can treat cancer-related bone pain cancer in children, and that it can be well tolerated when given subcutaneously. The use of subcutaneous clodronate may be useful in other conditions, such as osteogenesis imperfecta, where children receive bisphosphonates routinely and may express preferences for route of administration.

Another observation pertains to the widespread problem affecting much of Pediatrics in dealing with off-label medication uses [11]. The problem is compounded in pediatric palliative care where there is a critical dearth of evidence. In addition to trying to determine what medications can be safely and effectively used in children, clinicians must also decide the dose and route of administration. This report specifically described in detail the thought processes in developing a regimen for our patient in order to highlight these inter-related issues and demonstrate how they manifest in clinical situations.

This case report also demonstrates how a drug begins to be used in adults and then "shifts and drifts" (to borrow an epidemiology term), to use in children. The recommendation to simply conduct more pediatric clinical trials is a laudable one. It raises, however, the many difficulties of conducting studies involving children with rare diseases. There are many hurdles involved in pediatric studies – recruitment, consent and achieving statistical power in small populations.

One answer is for clinicians to develop the multi-centre networks required to answer questions regarding rare childhood conditions, especially at the end-of-life. A second is a reminder that when pediatric randomized trials are developed investigators should challenge themselves and ethical review boards to consider optimal designs, using randomized placebo-controlled trials where possible. A third suggestion is to consider the benefit of conducting and reporting n-of-1 trials. N-of-1 trials, which are single person, double-masked trials, can be combined to provide evidence of therapy. Lastly, when reporting new uses of old agents, authors should specifically demonstrate how they developed regimens even if based on weak evidence. This clarity will help other clinicians and researchers think more critically about developing further avenues for treatment and investigation.

In conclusion this case report describes the use of subcutaneous clodronate for analgesia in a child with a bone tumour, with good results. It also describes the challenges and thought processes that clinicians frequently encounter in devising new empirical treatments for children.

## Competing interests

The author(s) declare that they have no competing interests.

## Authors' contributions

**HS **provided clinical care to the patient and wrote the manuscript.
